# Micropipette-based biomechanical nanotools on living cells

**DOI:** 10.1007/s00249-021-01587-5

**Published:** 2022-02-16

**Authors:** Haoqing Wang, Fang Zhou, Yuze Guo, Lining Arnold Ju

**Affiliations:** 1 School of Biomedical Engineering, Faculty of Engineering, The University of Sydney, Darlington, NSW Australia; 2 Charles Perkins Centre, The University of Sydney, Camperdown, NSW Australia; 3 Heart Research Institute, Newtown, NSW Australia

**Keywords:** Dynamic force spectroscopy, Micropipette, Cortical tension, Mechanobiology

## Abstract

Mechanobiology is an emerging field at the interface of biology and mechanics, investigating the roles of mechanical forces within biomolecules, organelles, cells, and tissues. As a highlight, the recent advances of micropipette-based aspiration assays and dynamic force spectroscopies such as biomembrane force probe (BFP) provide unprecedented mechanobiological insights with excellent live-cell compatibility. In their classic applications, these assays measure force-dependent ligand–receptor-binding kinetics, protein conformational changes, and cellular mechanical properties such as cortical tension and stiffness. In recent years, when combined with advanced microscopies in high spatial and temporal resolutions, these biomechanical nanotools enable characterization of receptor-mediated cell mechanosensing and subsequent organelle behaviors at single-cellular and molecular level. In this review, we summarize the latest developments of these assays for live-cell mechanobiology studies. We also provide perspectives on their future upgrades with multimodal integration and high-throughput capability.

## Introduction

Mechanical milieu such as tensile force, fluid shear stress, compression, and substrate stiffness are increasingly recognized for a critical role in dynamic cellular behaviors including adhesion, migration, and differentiation. For the past decade, micropipette-based aspiration assays have been applied to measuring mechanical properties of cells such as elastic modulus, stiffness, and membrane tension (Gonzalez-Bermudez et al. 2019; Mierke 2021). On one hand, with finely fashioned orifice, the micropipette generates negative pressure that aspirates single cells (Chen et al. 2019; Husson et al. 2011; Swift et al. 2013), spheroids (Blumlein et al. 2017), and microtissues (Guevorkian and Maitre 2017). At the molecular level, dynamic force spectroscopies (DFS) have been developed to interrogate protein dynamics, particularly force-dependent binding kinetics and conformational changes (Dulin et al. 2015; Liu et al. 2015; Ungai-Salanki et al. 2019). The majority of these classical studies were conducted on purified molecular constructs or isolated cellular components (Carrion-Vazquez et al. 2000; Et-Thakafy et al. 2017; Ju et al. 2013).

As an emerging trend in recent years, technical integration such as the combined live-cell micropipette aspiration and DFS such as BFP (Chen et al. 2019; Husson et al. 2011; Ju et al. 2016; Liu et al. 2014; Wu et al. 2019) have enabled in situ investigation into cellular and molecular behaviors. The further upgrade with concurrent fluorescence microscopy provides new insights into receptor-mediated bi-directional signal transduction in response to mechanical micro-environment (Arbore et al. 2019; Zhu et al. 2019). For example, the binding kinetics and conformational changes on mechanoreceptors can be correlated with the triggered downstream intracellular signaling simultaneously (Ju et al. 2016; Liu et al. 2014). To the scope of this review, we will focus on the recent advancements of BFP and equivalent micropipette-based ultrasensitive force probe techniques in the context of single cell mechanosensing.

## Micropipette-based assays

### Single micropipette aspiration assays

Micropipette techniques are first used for live-cell microinjection, in vitro fertilization (Hiraoka and Kitamura 2015; Temple-Smith et al. 1985), and more recently, gene and genome editing (Rasys et al. 2019; Shao et al. 2014) (Table 1, 1st row). In a typical application, negative pressure is generated within an open-orifice micropipette to aspirate a single cell, such as an egg of sea urchins (Mitchison and Swann 1954), leukocytes (Lichtman 1973), red blood cell (RBC) (Jay 1973), and platelet (White et al. 1984) to investigates their responses to mechanical stimuli.

**Table 1:**
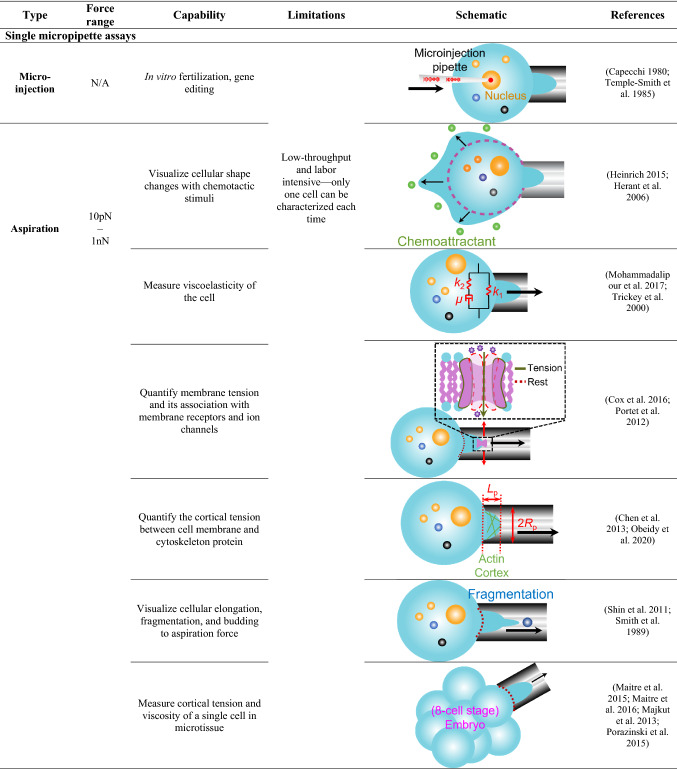

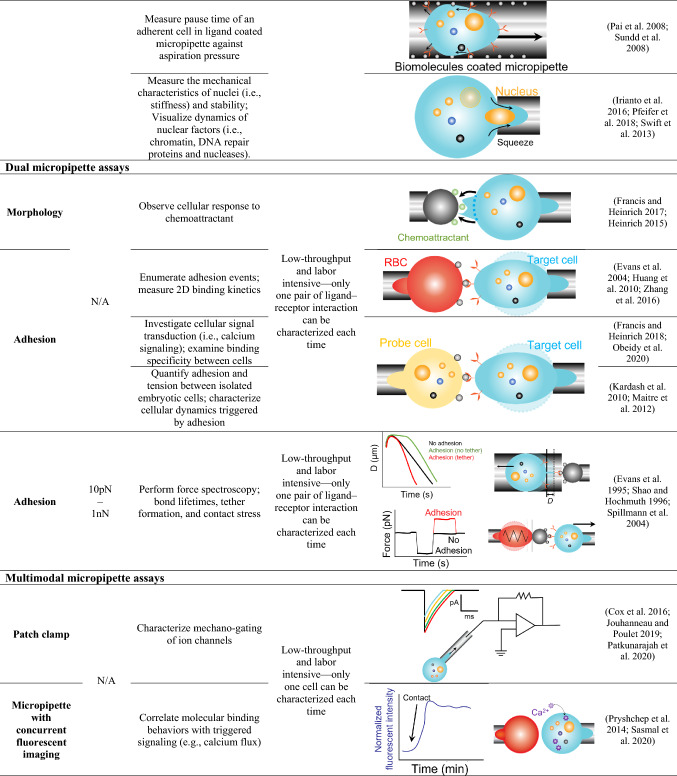

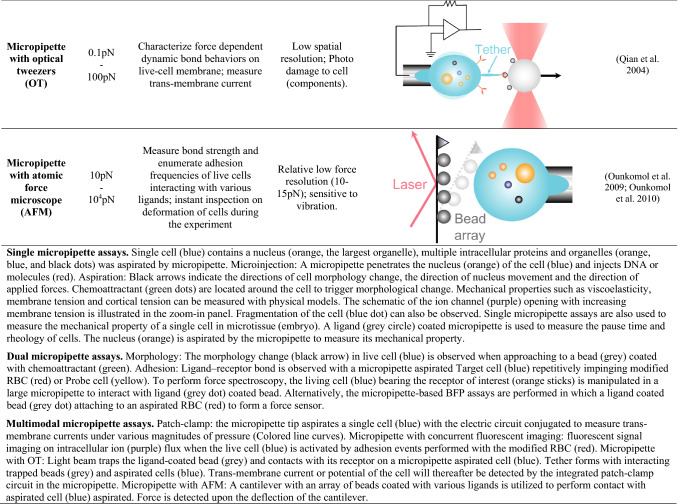
Micropipette-based cell mechanobiology applications

*Single micropipette assays* Single cell (blue) contains a nucleus (orange, the largest organelle), multiple intracellular proteins and organelles (orange, blue and black dots) was aspirated by micropipette. Microinjection: A micropipette penetrates the nucleus (orange) of the cell (blue) and injects DNA or molecules (red). Aspiration: Black arrows indicate the directions of cell morphology change, the direction of nucleus movement and the direction of applied forces. Chemoattractant (green dots) are located around the cell to trigger morphological change. Mechanical properties such as viscoelasticity, membrane tension and cortical tension can be measured with physical models. The schematic of the ion channel (purple) opening with increasing membrane tension is illustrated in the zoom-in panel. Fragmentation of the cell (blue dot) can also be observed. Single micropipette assays are also used to measure the mechanical property of a single cell in microtissue (embryo). A ligand (grey circle) coated micropipette is used to measure the pause time and rheology of cells. The nucleus (orange) is aspirated by the micropipette to measure its mechanical property.

*Dual micropipette assays* Morphology: The morphology change (black arrow) in live cell (blue) is observed when approaching a bead (grey) coated with chemoattractant (green). Adhesion: Ligand–receptor bond is observed with a micropipette-aspirated Target cell (blue) repetitively impinging modified RBC (red) or Probe cell (yellow). To perform force spectroscopy, the living cell (blue) bearing the receptor of interest (orange sticks) is manipulated in a large micropipette to interact with ligand (grey dot) coated bead. Alternatively, the micropipette-based BFP assays are performed in which a ligand-coated bead (grey dot) attaching to an aspirated RBC (red) to form a force sensor.

*Multimodal micropipette assays* Patch-clamp: the micropipette tip aspirates a single cell (blue) with the electric circuit conjugated to measure transmembrane currents under various magnitudes of pressure (Colored line curves). Micropipette with concurrent fluorescent imaging: fluorescent signal imaging on intracellular ion (purple) flux when the live cell (blue) is activated by adhesion events performed with the modified RBC (red). Micropipette with OT: Light beam traps the ligand-coated bead (grey) and contacts with its receptor on a micropipette-aspirated cell (blue). Tether forms with interacting trapped beads (grey) and aspirated cells (blue). Transmembrane current or potential of the cell will thereafter be detected by the integrated patch-clamp circuit in the micropipette. Micropipette with AFM: A cantilever with an array of beads coated with various ligands is utilized to perform contact with aspirated cell (blue) aspirated. Force is detected upon the deflection of the cantilever.

**Fig. 1:**
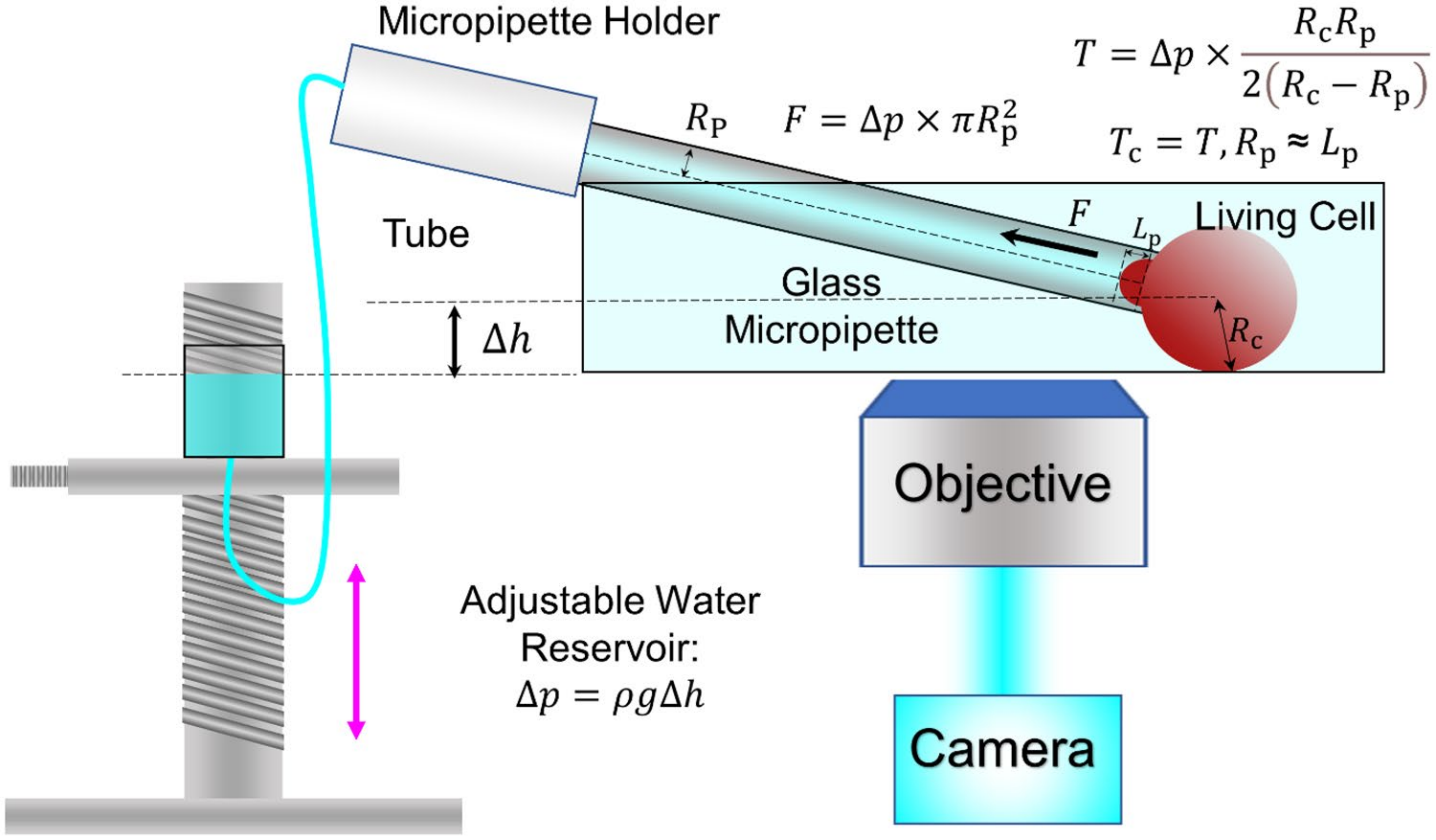
Schematic of micropipette-based aspiration assay. A borosilicate micropipette is used to aspirate a living cell (red) with negative pressure. The end of the micropipette holder is connected to a water reservoir whose height can be adjusted to precisely control the aspiration pressure. The cell behaviors are visualized with an inverted microscope

The borosilicate micropipette is connected to a reservoir with a micromanipulator that applies negative pressure in a stepwise manner (Fig. 1). The piconewton level force *F* (10 pN–1 nN, Table 1) on an aspirated cell (termed “Target”) is given by

1 $$F = \Delta p \times \pi {R_{{\text{p}}}^{2}} ,$$

where *R*_p_ is the radius of the micropipette orifice and Δ*p* is the aspiration pressure manipulated by adjusting the height of the reservoir (Hochmuth 2000).

2 $$\Delta p = \rho g\Delta h.$$

Controlled aspiration pressure prevents over-constrictive manipulation induced physical damage or pre-activation of the target cell. It is crucial to maintain the water level in the reservoir such that the aspiration pressure is kept constant during the experiment. Whenever the water level changes, it is important to re-calibrate the zero pressure thereby ensuring the accuracy of suction pressure (Ju et al. 2017b).

The single micropipette aspiration assays enable real-time cellular observations on morphological changes (Heinrich 2015; Herant et al. 2006) (Table 1, 2nd row), and measurement of mechanical properties such as viscoelasticity (Mohammadalipour et al. 2017; Trickey et al. 2000) (Table 1, 3rd row) and membrane tension (Cox et al. 2016; Portet et al. 2012) (Table 1, 4th row).

The cellular membrane tension *T* measured in the single micropipette aspiration assay is given by:

3 $$T = \Delta p \times \frac{{R_{{\text{c}}} R_{{\text{p}}} }}{{2\left( {R_{{\text{c}}} - R_{{\text{p}}} } \right)}},$$

where *R*_c_ is the radius of the aspirated target cell (Fig. 1). Portet *et al*. used the technique to validate the correlation between membrane tension and miscibility temperature and provide more insights on how membrane tension regulates the conformation of lipid bilayers (Portet et al. 2012). Meanwhile, Cox *et al*. showed that applying larger membrane tension via aspiration increased open possibility of the mechanosensitive ion channel Piezo1 (Cox et al. 2016). The finding on Piezo1 being gated by membrane tension supports the ‘Force-From-Lipids’ principle applied to Piezo channels.

When ∆*p* in Eq. 3 is adjusted to ensure the tongue length of the target cell *L*_p_ (aspirated cell portion inside the micropipette) is equal to *R*_p_, the cortical tension of the cell, *T*_c_ can thereafter be quantified (Hochmuth 2000) (Fig. 1 and Table1, 5th row).

4 $$T_c=T, \,R_p\,\approx\,L_p$$

Given that cortical tension is mediated by the connection between the cell membrane and the actin cytoskeleton in the cortex, the stability of membrane–cytoskeleton linkage in megakaryocytes and lymphocytes can be described and quantitated (Chen et al. 2013; Obeidy et al. 2020). Moreover, the single micropipette aspiration assays were also used to characterize the membrane fragmentation, elongation, and budding (Table1, 6th row). As an example, the preset pressure applies to a megakaryocyte aspirated by a micropipette in order to observe platelet generation (Shin et al. 2011; Smith et al. 1989). This micropipette model mimics the constrictive effect of blood flow during thrombopoiesis nicely. Furthermore, the single micropipette assays can also be applied to microtissues (Table 1, 7th row). For example, a micropipette was used to aspirate an eight-cell stage mouse embryo (Maitre et al. 2015). The cortical tension measurement by micropipettes helped define a new role of actomyosin in generating the compaction and initiating morphogenesis. Amazingly, the micropipette aspiration assays also demonstrated that cortical tension affects cell positioning and fate specification when blastomeres self-organize into a blastocyst (Maitre et al. 2016). A similar system is also used to measure the viscoelasticity of embryonic microtissues (Majkut et al. 2013; Porazinski et al. 2015).

Single micropipettes have also been used as a microfluidic channel to measure the micro-rheology of cells (Table1, 8th row). When the cell is being aspirated into a ligand-coated micropipette, the suction force applied to the cell is given by:

5 $$F = \Delta p\pi {R_{{\text{p}}}^{2}} \left( {1 - \frac{{U_{{\text{t}}} }}{{U_{{\text{f}}} }}} \right){, }$$

where *U*_t_ is the velocity of an interacting cell and *U*_f_ is the velocity of a free-moving cell (Shao and Hochmuth 1997). In a similar context, some studies also selectively coated P-selectin on the inner lumen of a micropipette and measured the neutrophil resistant time when applying negative pressure (Pai et al. 2008; Sundd et al. 2008).

Using micropipette aspiration to measure the nuclear stiffness and characterize the nuclear stability represent emerging application to intracellular mechanobiology (Table1, 9th row). Lamins, which form a dense protein network in the inner nuclear membrane, play a critical role in nucleus mechanosensing (Ho and Lammerding 2012). Swift *et al*. established micropipette assays to aspirate human lung-derived A549 cells with fluorescent labeled nuclear lamins and characterized the mechanical property of the nucleus (Swift et al. 2013). The results revealed that the level of lamin A, which contributes to lineage determination of the stem cell, is scaled with the nuclear stiffness. In addition, cells can be squeezed when moving through three-dimensional tissue. With micropipette aspiration assays, Irianto *et al*. and Pfeifer *et al*. observed mechanosensing dynamics of DNA repair proteins and nucleases when being squeezed into the micropipette (Irianto et al. 2017; Pfeifer et al. 2018). Results demonstrated that extracellular pressure from the trans-tissue migration will cause the intranuclear chromatin and DNA damage of cancer cells. The dynamic of the nuclei illustrates the ‘go, damage and grow’ behavior of cancer cells.

### Dual micropipette assays

Adding an apposing micropipette, dual micropipette assays can characterize cell–cell or cell–molecule interaction and associated biophysical parameters with controlled engagement or separation (Biro and Maitre 2015; Ju et al. 2017b). As an example, the dual micropipette assay was used to present fungi or bacteria induced chemotaxis on neutrophils (Table 1, 10th row), which quantifies the sensitivity of immune cells to chemoattractant (Francis and Heinrich 2017; Heinrich 2015). The dual micropipette adhesion frequency assays have also been developed to measure ligand–receptor-binding kinetics in two dimensions, or 2D kinetics (Evans et al. 2004; Huang et al. 2010; Zhang et al. 2016) (Table 1, 11th row). In such system, RBCs and beads were functionalized with the proteins of interest via covalent bonding. The adhesion frequency *P*_a_ is enumerated by pinches of RBC cell membrane during repetitive cell–cell touches (Piper et al. 1998) then derive into 2D on- (*k*_on_) and off-rates (*k*_off_) by a probabilistic model given by

6 $$P_{{\text{a}}} = 1 - {\text{exp}}\left\{ {m_{{\text{r}}} m_{{\text{l}}} A_{{\text{c}}} k_{{{\text{on}}}} \left[ {1 - \exp \left( { - k_{{{\text{off}}}} t_{{\text{c}}} } \right)} \right]/k_{{{\text{off}}}} } \right\},$$

where *m*_r_ and *m*_l_ are the respective receptor and ligand densities, *t*_c_ is the contact time, and *A*_c_ is the contact area of two cells (Ju et al. 2017b).

Meanwhile, the dual micropipette has also been implemented to investigate the adhesive behavior of cell–cell interaction (Table 1, 12th row). For example, Obeidy *et al.* revealed that the modulation on actin-related protein 2/3 complex would reduce the filamentous F-actin formation and cytotoxicity in primary T lymphocytes (Obeidy et al. 2020). The resulting insights into T-cell migration and function inspired alternative treatment for cancer and inflammatory disease. Furthermore, studies also employed a dual micropipette system to investigate the adhesive behaviors of individual microtissue cells from zebrafish embryos (Table 1, 13th row). Maitre *et al.* showed that embryo cell adhesion is mediated by E-cadherin, which provides the mechanical scaffold for cortical tension to modulate cell sorting during gastrulation (Maitre et al. 2012). With similar assays, Kardash *et al.* demonstrated that the motility of chemokine-guided germ cells needs the function of Rho GTPases and E-cadherin-mediated-adhesion (Kardash et al. 2010). Both two studies provide more insights into stem-cell differentiation and migration processes.

### Multimodal micropipette assays

Micropipette techniques have been further upgraded with multimodalities by combining with external physical electrical, optical and mechanical fields. By integrating an electric circuit of patch clamping, micropipettes have been used to identify mechanosensitive ion channels such as Piezo1 (Cox et al. 2016; Patkunarajah et al. 2020), TRPV4 (Servin-Vences et al. 2017), MscL (Moe and Blount 2005), and characterize their mechano-gating kinetics (Table 1, 14th row). Similarly, by adding a fluorescent light path, the micropipette assays correlated cellular adhesive behaviors with triggered calcium mobilization (Heinrich 2015; Pryshchep et al. 2014) and other intracellular signaling responses (Sasmal et al. 2020) (Table 1, 15th row). Moreover, researchers have combined the micropipette aspiration assays with patch clamping and optical tweezers (OT) setups to study membrane electromechanical properties (Table 1, 16th row). This integrated system can pull cell membrane tethers, measure the cytoskeletal disruption force then correlate with the transmembrane potential (Qian et al. 2004). Ounkomol *et al.* also combined a micropipette system with atomic force microscopy (AFM) to apply compressive and tensile force horizontally and more importantly, the side view of the experiment provides optical feedback to correct for drifts in longtime experiments (Ounkomol et al. 2009) (Table 1, 17th row). With a unique assembly of ligand coated bead arrays on a commercial cantilever, the hybrid system is capable of interrogating interactions of the same cell with multiple ligand species (Ounkomol et al. 2010). The study clarifies that E- and N-cadherins can readily form specific heterophilic bonds, but less frequently than homophilic bonds of either cadherin.

## Dynamic force spectroscopy

**Fig. 2:**
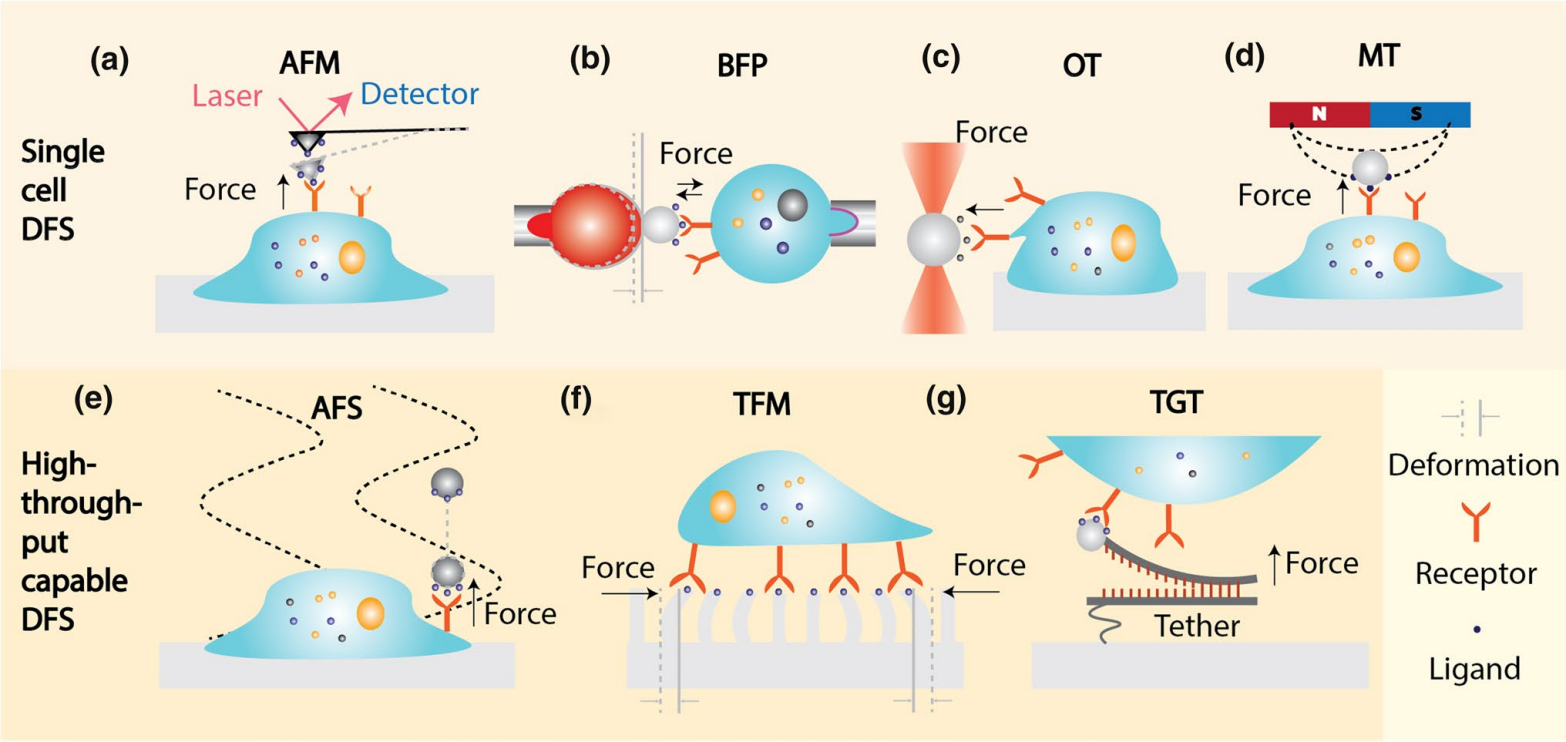
Representative dynamic force spectroscopies on living cells. **a-d** Single-cell DFS assays. **a** Atomic force microscopy (AFM). A ligand-coated cantilever is utilized to scan a spreading cell and form contact with its surface receptor. Force is derived from the deflection of the cantilever beam through a detector. **b** Biomembrane force probe (BFP). A ligand-coated bead is glued on the apex of treated red blood cell and then, ligand–receptor bond is characterized by repetitive touch cycles. Force is detected from the deflection of red blood cell. **c** Optical tweezers (OT). A laser beam is applied to trap the ligand-coated bead or live cell for manipulating the cell–cell interaction and single molecular binding. The distance between the trapped bead/cell and the focus of the laser is measured to calculate the force. **d** Magnetic tweezers (MT). A protein-coated bead is controlled by the gradient of the magnetic field and the motion of the bead is tracked. The exerted force is proportional to the direction toward the strongest magnetic field and the gradient of the applied magnetic field. **e–g** High-throughput capable DFS assays. **e** Acoustic Force Spectroscopy (AFS). Ligand-coated beads are driven away from attached cells by applying an acoustic filed. Displacement of the beads is tracked in real time. The physical model allows to derive force in the function of applied acoustic amplitude. **f** Traction force microscopy (TFM). Cells are spreaded on the matrix through specific ligand–receptor bonds. Deformations on micropillars are measured to calculate the cell traction force. **g** Tension Gauge Tether (TGT). A double strand DNA is utilized with one strand attaching to the surface and the other strand conjugated with ligands bind to a receptor on the live cell. The double-stranded DNA is designed to split once rupture force is reached where the rupture force is tuned by the sequence and length of double-stranded DNA

DFS techniques are widely used to manipulate, characterize, and visualize the force-dependent binding kinetics with live cells, as well as to investigate protein conformational changes. Seven representative DFS are categorized as ‘Single-cell’ (Fig. 2a–d) or ‘High-throughput capable’ (Fig. 2e–g). Automated precise movement brings ligand-coated force probe (i.e., cantilever in Fig. 2a, beads in Fig. 2b–e and g, and micropillars in Fig. 2f) together with receptor-bearing live cells with controlled force, time, and area. Then, the contacted surface will be applied with piconewton force to modulate the ligand–receptor bond dissociation. Each DFS technique has its distinctive rationale of force application and measurement. Single-cell DFS assays including AFM (Fig. 2a), micropipette-based BFP (Fig. 2b), OT (Fig. 2c), and Magnetic Tweezers (MT; Fig. 2d), measure molecular binding forces one cell at a time (Su and Ju 2018). AFM utilizes a ligand-coated cantilever beam to exert force on a cell that usually spreads on a substrate matrix. The molecular binding force is measured by monitoring the cantilever deflection with a photodiode. Cellular properties, including topography and stiffness, are then evaluated. BFP uses a micropipette to gently aspirate the cell in its native state without pre-activation (Fig. 2b), making this technique compatible with certain mechanosensitive-cell types such as platelets (Ju et al. 2016) and primary T-cells (Liu et al. 2014). OT applies a focal laser beam to generate trapping force and mechanically manipulate beads and cells (Fig. 2c). Comparing to the OT, MT utilizes a gradient magnetic field to manipulate beads and cells to perform controlled interactions (Fig. 2d).

To achieve high-content DFS measurements, acoustic force spectroscopy (AFS, Fig. 2e) was invented to manipulate multiple bead–cell pairs simultaneously with an applied acoustic force field (Romanov et al. 2021). AFS has recently been used to pull membrane tethers on tens to hundreds of cells and measure their viscoelastic properties in a high-throughput manner. In a similar token, traction force microscopy (TFM, Fig. 2f) utilizes bead embedded gel or flexible micro-pillar arrays of 2–20 µm sizes to measure traction force generated during cell adhesion and migration. A more recent advance of soft substrates produced by electron beam lithography enables visualization of cellular mechanosensing at a submicron resolution (Ghassemi et al. 2012; Hanson et al. 2015). Last but not least, double-stranded DNA has been repurposed to measure rupture forces of specific ligand–receptor bonds, termed ‘tension gauge tether’ (TGT, Fig. 2g). The force threshold is tuned by the DNA sequence and length. The upper DNA strand is linked with ligands and binds to corresponding receptors on cells (Jo et al. 2019; Wang 2013; Zhang et al. 2018). In this review, micropipette-based DFS, BFP and its equivalent micropipette-based ultrasensitive force probe techniques will be discussed as their rapid development on live cells manipulation.

## Biomembrane force probe (BFP)

**Fig. 3:**
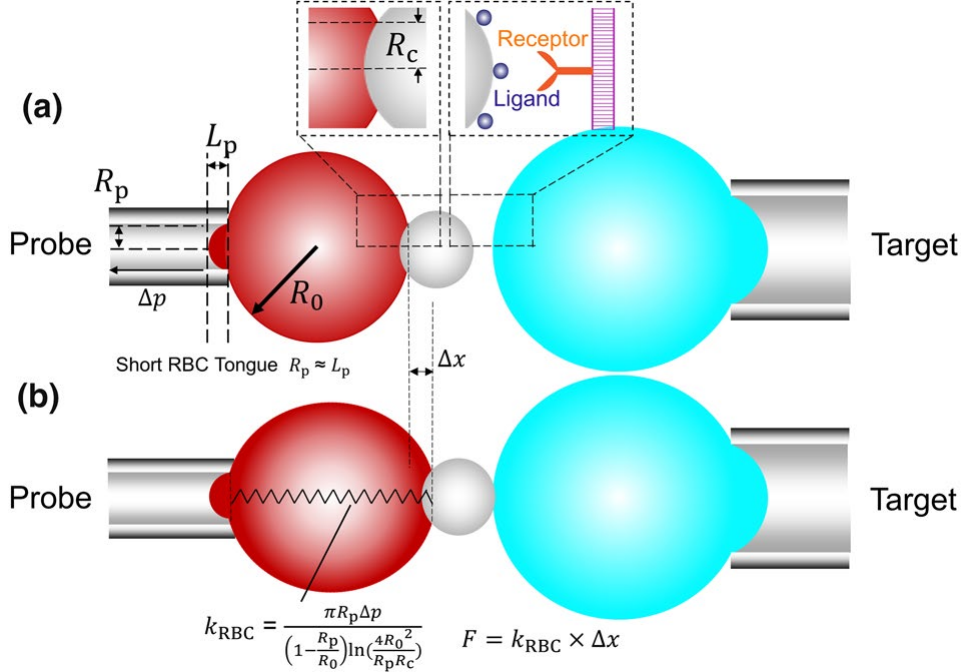
Schematics of biomembrane force probe and its force measurement rationale. **a** A micropipette-aspirated RBC with a bead (left, termed “Probe”) attached to the apex formed a pico-force spring sensor, as depicted by a spring. It was aligned with the living cell aspirated by an apposing micropipette (right, termed “Target”). **b** The edge of RBC and Probe bead was tracked by valley detection algorithm in the program and then holding force can be derived by Hooke’s law

BFP was first introduced by Evans et al. (1995) and is thereafter widely implemented to characterize molecular bonds between various proteins. The technique represents an upgraded dual micropipette assay (Fig. 2b) and has become one of the emerging live-cell DFS techniques to interrogate the biomechanical regulation of two-dimensional (2D) ligand–receptor-binding kinetics (Chen et al. 2019; Liu et al. 2014). Compared to the dual micropipette system using beads to measure bond lifetime, BFP has advantages in force, temporal, and spatial resolution. Both purified molecules and molecules on living cells are involved in BFP. Prior to experiments, an RBC is pre-swollen and aspirated by the Probe micropipette with a ligand-coated bead attached to its apex to form a piconewton force sensor (Fig. 3a). The Spring constant of the RBC (*k*_RBC_) is calculated from the magnitude of pressure applied and the radii of the orifice (*R*_p_), the Probe bead (*R*_c_) and RBC (*R*_0_) itself if the radius of the orifice (*R*_p_) is equal to the aspirated RBC length (*L*_p_):

7 $$k_{{{\text{RBC}}}} = \frac{{\pi R_{{\text{p}}} \Delta p}}{{\left( {1 - \frac{{R_{{\text{p}}} }}{{R_{0} }}} \right)\ln \left( {\frac{{4R_{0}^{2} }}{{R_{{\text{p}}} R_{{\text{c}}} }}} \right)}}, L_{{\text{p}}} \approx R_{{\text{p}}} .$$

Normally, the spring constant will be set to 0.25 or 0.3 pN/nm (Ju and Zhu 2017). The apposing Target micropipette aspirates a receptor-bearing living cell and is then driven by a Piezo actuator to impinge the Probe. Then, the sub-piconewton force *F* exerted by Target is detected by the deflection of the RBC (Δ*x*, Fig. 3b) based on Hooke’s Law:

8 $$F = k_{{{\text{RBC}}}} \times \Delta x.$$

With repetitive Probe–Target touch cycles, preset piconewton force (–10^3^ pN, Table 2) is applied onto the ligand–receptor bond. The molecular binding dynamics are visualized and depicted by the force spectroscopies in real time. During the experiment, it is assumed that the morphology of the RBC is not dramatically changing over time, otherwise, a fresh RBC needs to be replaced to ensure force accuracy (Ju and Zhu 2017).

**Table 2:**
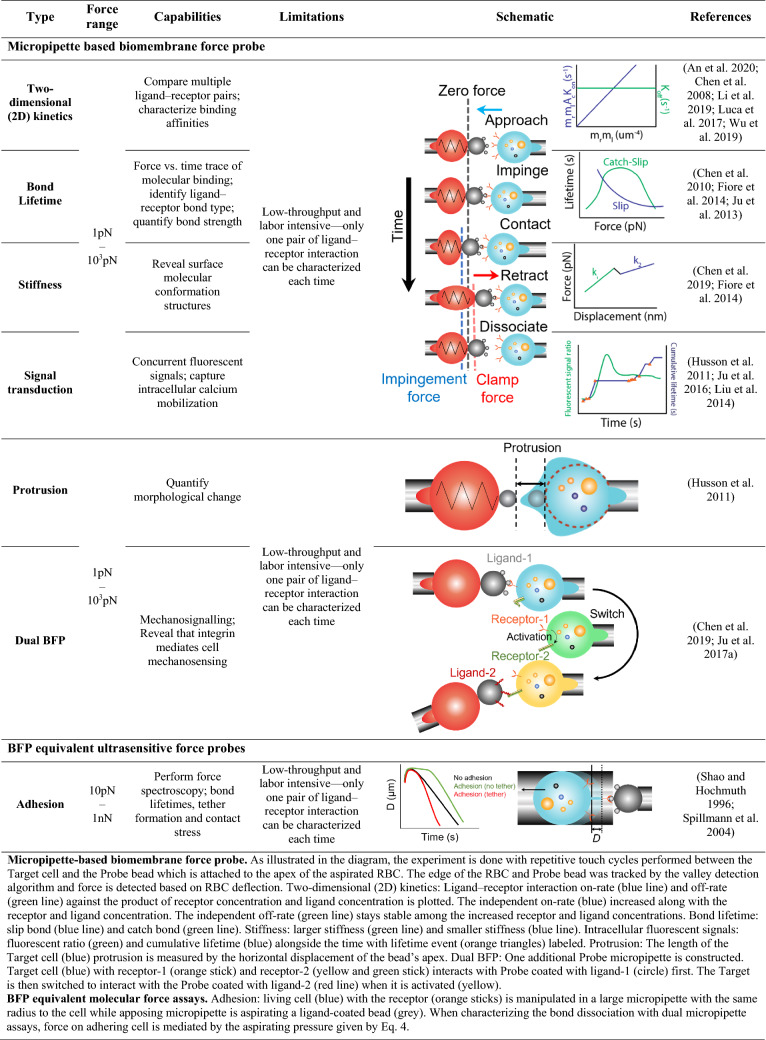
BFP and equivalent biophysical applications

*Micropipette-based biomembrane force probe* As illustrated in the diagram, the experiment is done with repetitive touch cycles performed between the Target cell and the Probe bead which is attached to the apex of the aspirated RBC. The edge of the RBC and Probe bead was tracked by  the valley detection algorithm and force is detected based on RBC deflection. Two-dimensional (2D) kinetics: Ligand–receptor interaction on-rate (blue line) and off-rate (green line) against the product of receptor concentration and ligand concentration is plotted. The independent on-rate (blue) increased along with the receptor and ligand concentration. The independent off-rate (green line) stays stable among the increased receptor and ligand concentrations. Bond lifetime: slip bond (blue line) and catch bond (green line). Stiffness: larger stiffness (green line) and smaller stiffness (blue line). Intracellular fluorescent signals: fluorescent ratio (green) and cumulative lifetime (blue) alongside the time with lifetime event (orange triangles) labeled. Protrusion: The length of the Target cell (blue) protrusion is measured by the horizontal displacement of the bead’s apex. Dual BFP: One additional Probe micropipette is constructed. Target cell (blue) with receptor-1 (orange stick) and receptor-2 (yellow and green stick) interacts with Probe coated with ligand-1 (circle) first. The Target is then switched to interact with the Probe coated with ligand-2 (red line) when it is activated (yellow)

*BFP equivalent molecular force assays* Adhesion: living cell (blue) with the receptor (orange sticks) is manipulated in a large micropipette with the same radius to the cell, while apposing micropipette is aspirating a ligand-coated bead (grey). When characterizing the bond dissociation with dual micropipette assays, force on adhering cell is mediated by the aspirating pressure given by Eq. 4

### Dynamic bond measurement

BFP offers multiple analysis modes (Table 2) including thermal fluctuation (Chen et al. 2008), force-ramp assay, and force-clamp assay (Chen et al. 2017) to measure single bond 2D kinetics, in terms of association (Chen et al. 2008; Li et al. 2019; Luca et al. 2017) and dissociation rate (An et al. 2020; Wu et al. 2019) (Table 2, 1st row), bond lifetime (Chen et al. 2010; Fiore et al. 2014; Ju et al. 2013), and bond stiffness (Chen et al. 2019; Fiore et al. 2014). In the force-clamp assay, BFP is able to measure the bond lifetime of specific ligand–receptor bonds over a range of forces (Table 2, 2nd row). The force vs. bond lifetime curves thereafter reflects the interaction as one of two bond types: slip bond or catch bond. A slip bond has a decreased lifetime along with the rising clamp force, demonstrating a force-weaken interaction, whereas a catch bond has an increased lifetime, demonstrating a force-strengthen interaction. With this force-clamp assay, studies reveal how integrin mediates cell–cell adhesion in a dynamic environment such as cancer cell and endothelial cell (Fiore et al. 2014), leukocyte (Chen et al. 2010), and von Willebrand factor (VWF) and platelets (Ju et al. 2013). Chen *et al.* applied BFP to discover the catch-slip bond formed between integrin α_L_β_2_, or lymphocyte function associated antigen-1 (LFA-1), and intercellular adhesion molecule-1 (ICAM-1) (Chen et al. 2010). They further revealed the internal catch bond between αA and βA domains in α_L_β_2_ would allosterically affect its binding affinity with ICAM-1. Ju *et al.* used force clamp DFS in BFP to characterize the catch bond between the A1 domain of VWF and platelets, which provide more structural insight into VWF activation by hemodynamic force of circulation (Ju et al. 2013).

Furthermore, the RBC, the target cell, and ligand–receptor bond in the BFP system can be considered as serially connected springs when stretched. Bond stiffness can be derived from the DFS data to subsequently depict the distinct conformation status of the complex (Chen et al. 2019; Fiore et al. 2014) (Table 2, 3rd row). For example, BFP was applied to analyzing Thy-1–α_5_β_1_ interactions on K562 cells. Bond stiffness analysis was so critical that detected Thy-1 also interacted with syndecan-4 receptor to form a trimolecular complex with a catch-bond behavior (Fiore et al. 2014). The finding elucidated how Thy-1 on endothelial cells support the adhesion of cancer cells in a mechanically stressed environment. Besides, by analyzing bond stiffness, lifetime and 2D kinetics, Chen and Ju et al. identified a biomechanically activated intermediate state on platelet integrin αIIbβ3 (Chen et al. 2019). Taken together, these mechanobiology insights inspired new anti-thrombotic strategies (Chen and Ju 2020).

### Bi-directional receptor-mediated cell mechanosensing

Recent integrations of concurrent fluorescent imaging in BFP assays enabled new understanding on both ‘outside-in’ (Husson et al. 2011; Ju et al. 2016; Liu et al. 2014) and ‘inside-out’ (Chen et al. 2019; Ju et al. 2017a) cell mechanosensing pathways. Mechanical outside-in signaling describes the receptor-mediated mechanosensing upon the extracellular applied tensile and compressive forces, fluid shear, and transduction towards intracellular space (Chen et al. 2017; Zhu et al. 2019). Mechanical cues and milieu are converted into biological signals which trigger downstream intracellular events. When combined with concurrent fluorescence imaging, the BFP is able to provide real-time intracellular event recording alongside external force stimulation (Chen et al. 2015) (Table 2, 4th row). The most common cellular event along with force application is calcium flux, as calcium concentration plays a critical role in integrin activation. These fluorescent BFP assays have demonstrated tensile force triggered T-cell activation and cytotoxicity (Husson et al. 2011; Liu et al. 2014). The results revealed that activation of T-cell increased cell stiffness thereby resisted the protrusion (Husson et al. 2011) (Table 2, 5th row). Similarly, fluorescence BFP has been applied to platelet mechanobiology, elucidating their biomechanical activation and aggregation underlying arterial thrombosis (Ju et al. 2016).

On the reverse direction of the mechanical outside-in signaling pathway, the inside-out signaling pathway has recently been demonstrated by multiple BFP experiments and proposed as an emerging concept (Zhu et al. 2019). Inside-out mechanical signaling pathway describes that the intracellular forces, mostly from  the cytoskeleton, would allosterically affect endoplasmic and transmembrane domains of surface receptor proteins, further influencing their binding kinetics toward external ligands. In the inside-out signaling pathway, the intracellular forces are generated by a series of interconnected cytoplasmic proteins including filament actin, myosin, both of which support cell migration and adhesion. This inside-out signaling triggered allosteric deformation on transmembrane receptors is possibly ionic flux coupled. Further upgrade into a dual BFP system led to signal crosstalk studies between multiple mechanoreceptors i.e. the GPIbα mediated on inside-out activation of integrin α_IIb_β_3 _(Chen et al. 2019; Ju et al. 2017a) (Table 2, 6th row).

### BFP equivalent ultrasensitive force probes

There are also other equivalent force measurement assays to BFP. In dual micropipette assays, one of the micropipettes is fashioned into a radius equal to, or slightly smaller than the radius of the living cell. Such design can translate the living cell inside the micropipette to approach, forming contact, and then retract from the ligand-coated beads aspirated by the apposing micropipette (Table 1, 7th row). With adhesion formed, the clamp force is measured according to Eq. 4 (Shao and Hochmuth 1996). The method is able to quantify the tether formed between T-cell and its antibody IgG (Shao and Hochmuth 1996). Spillmann *et al.* use the same assay to reveal that the bond formation between β_2_-integrins on neutrophil and immobilized ICAM-1 is linearly increased with a larger contact area with fixed contact stress, but that increasing contact stress leads to higher rates of bond formation (Spillmann et al. 2004). Both of these two studies provide more insights into neutrophils binding dynamics on vascular walls in physiological conditions.

## Discussion

The comparative advantage of micropipette-based assays is that precisely controlled aspiration has controllable and subtle physical damage on living cells. As a result, this advantage enables long-period observation and characterization of mechanosensing behaviors on a variety of cell types. More significantly, such assays have minimized the pre-activation effect on primary cells such as platelets (Ju et al. 2016) and T-cells (Liu et al. 2014), which are naturally mechanosensitive. BFP, the upgraded dual micropipette assay, is a very powerful DFS nanotool to investigate ligand–receptor-binding kinetics with high temporal, and spatial resolution. The recent upgrade with concurrent fluorescent imaging (Chen et al. 2015) and dual BFP setup (Chen et al. 2019; Ju et al. 2017a) further enable the technique to characterize both the outside-in and inside-out mechanosensing (Zhu et al. 2019).

Amongst micropipette-based assays, it is a challenge to quantify intracellular forces due to cell complexity. Meanwhile, throughput is confined as only a single live cell can be manipulated simultaneously. A similar problem also exists amongst BFP and other equivalent assays. To gather sufficient data with statistical significance, the technique is very time-consuming as only one pair of ligand–receptor interaction or one type of intracellular ion flux can be characterized each time (Ju et al. 2017a) which creates the barrier to high-throughput (Chen et al. 2017).

Nevertheless, we still anticipate the rapid development of the aforementioned biomechanical nanotools and their applications with the following future perspective:

### Multimodality

The future technical upgrade should involve multimodal integration and combine strengths of micropipette and several DFS techniques to support more comprehensive biomechanical studies. In recent decades, there is an increasing number of assays using OT to trap ligand-coated beads with the target cell (Qian et al. 2004) or bead (Arce et al. 2021; Kim et al. 2010) aspirated on an apposing micropipette to perform force measurements. Micropipette-based assays are advantageous for probing membrane ligand–receptor dynamics with minimized cell drifting, whilst OT could reach intracellular protein and organelle. Thus, the combination of both techniques on live-cell and long-duration DFS experiments could provide more comprehensive understanding of binding dynamics and receptor-mediated cell mechanosensing. Another promising example is fluid force microscopy (FluidFM), which combines fluidic devices with AFM in which the cantilever beam was filled with pressure-controlled external fluid. This FluidFM has been applied to single-cell binding force quantification (Dorig et al. 2013; Potthoff et al. 2012).

### High throughput

Current biomechanical measurements on micropipettes and DFS are often performed one cell at a time. The sophisticated experimental procedures are usually labor intensive, leading to a steep learning curve. The recent development of automated aspiration with micropipette-based assays provides a four times faster manipulation velocity (Liu et al. 2019), which guides a pathway to improve the efficiency of current assays. Nevertheless, high-content DFS development is in great need to enhance experimental efficiency and statistical significance. Currently, AFS, TFM, and TGT are promising high-content DFS techniques with 30–50 cells that can be analyzed reliably (Ghassemi et al. 2012; Hanson et al. 2015; Jo et al. 2019; Romanov et al. 2021). However, there are still complementary techniques to the single cell DFS such as BFP and OT due to their bottlenecked force resolution and accuracy.

### Move inside the cell

Implementing micropipettes and DFS in understanding intracellular mechanobiology is also a topical area. For instance, the mechanoreceptors such as integrins on live cells transduce extracellular mechanical stimuli, and mediate intracellular signaling. When combined with fluorescence microscopy, DFS techniques are more powerful to characterize the mechanosignaling pathway in both mechanical outside-in and inside-out pathways (Roca-Cusachs et al. 2017; Zhu et al. 2019). Meanwhile, intracellular space contains a large number of adaptor proteins that further translate mechanical cues into biochemical signals and induce nucleus activities. To interrogate these, manipulation needs to happen inside the cell. DFS nanotools are focusing on improving their capabilities towards intracellular measurements. To this end, OT-DFS technologies have rapidly developed to detect intracellular mechanotransduction events at higher spatial and temporal resolutions (McCauley et al. 2019; Venturini et al. 2020). Recent advancement in MT setup also enables intracellular measurement with a higher force and extended working distance (Selvaggi et al. 2018). It is also anticipated that new intracellular protein tracers and force sensors to be developed and combined with DFS. Taken together, these perspective technical advances promise to unearth the unprecedented mechanobiology insights from outside to inside the cell.

## Data Availability

Not applicable. Not applicable.
